# The Application and Diagnostic Accuracy of Artificial Intelligence in Rhinology: A Review

**DOI:** 10.7759/cureus.87966

**Published:** 2025-07-15

**Authors:** Shrikrishna B.H., Deepa G.

**Affiliations:** 1 Otorhinolaryngology Head-Neck Surgery, All India Institute of Medical Sciences, Bibinagar, Hyderabad, IND; 2 Anatomy, All India Institute of Medical Sciences, Bibinagar, Hyderabad, IND

**Keywords:** artificial intelligence, deep learning, diagnostic imaging, machine learning, rhinology

## Abstract

Artificial intelligence (AI) technologies, including machine learning (ML), deep learning, and large language models, are increasingly applied in medical diagnostics. In rhinology, these tools are being evaluated for tasks such as image interpretation, cytology classification, and clinical decision support. To systematically evaluate the application and diagnostic accuracy of AI technologies in rhinology, with a focus on clinical utility and implementation barriers. This review followed Preferred Reporting Items for Systematic Reviews and Meta-Analyses (PRISMA) 2020 guidelines. Seventeen full-text studies were screened based on predefined eligibility criteria, focusing on AI applications with diagnostic metrics in rhinology. Data on AI type, diagnostic task, performance outcomes, and study quality were extracted and synthesized narratively. Twelve studies met the inclusion criteria. Image-based diagnostic tools using convolutional neural networks demonstrated high accuracy (81%-99%) in nasal polyp detection, cytology classification, and computed tomography (CT) scan interpretation. ML models using patient-reported data achieved accuracies of 74.5%-85.5% for chronic rhinosinusitis prediction. Large language models like ChatGPT and Gemini were evaluated for clinical question answering, with performance exceeding 80% in some domains. Risk of bias was moderate in most primary studies, and none reported clinical integration beyond prototype stages. AI exhibits promising diagnostic accuracy across several applications in rhinology. However, significant challenges persist, including limited validation, methodological heterogeneity, and lack of clinical implementation. Future research should focus on prospective trials, explainability, and regulatory frameworks to ensure safe integration into clinical workflows.

## Introduction and background

Artificial intelligence (AI) has rapidly permeated various fields of medicine, including radiology, pathology, and, more recently, otolaryngology. Within the subdomain of rhinology, AI-driven technologies, such as machine learning (ML), deep learning (DL), convolutional neural networks (CNNs), and large language models, are being applied to assist in diagnostic processes, optimize image interpretation, and support clinical decision-making. AI applications in rhinology predominantly revolve around image analysis in endoscopy and radiology, cytological classification, prediction of disease outcomes, and automated clinical question answering. Studies have demonstrated the potential of CNN-based models to detect nasal polyps with 98.3% accuracy [1], concha bullosa from computed tomography (CT) scans with 81% accuracy [2], and nasal cytology classification models achieving up to 99% test accuracy [3]. ML models trained on patient-reported outcome measures have shown promise in predicting chronic rhinosinusitis with accuracies ranging from 74.5% to 85.5%, albeit with lower sensitivities [4]. Radiomics applications in paranasal sinus CT have yielded variable diagnostic accuracies (area under the receiver operating characteristic (ROC) curve (AUC) 0.73-0.92) [5], while large language models like ChatGPT have been evaluated for clinical question answering with over 80% accuracy in guideline adherence assessments [6,7]. Despite the growing interest, most studies remain at the prototype or proof-of-concept stage, and none have been validated for routine clinical use. This review aims to evaluate the diagnostic accuracy and application domains of AI in rhinology and provide insights into current limitations and future research needs.

## Review

Methodology

Eligibility Criteria

Studies were eligible if they met the following criteria: (1) included human subjects or clinically relevant datasets; (2) examined AI applications for diagnostic purposes in rhinology; (3) reported quantifiable diagnostic performance (e.g., accuracy, sensitivity, specificity, AUC); (4) were full-text original research articles or reviews; and (5) involved ≥10 samples or patients. Only studies published in English were considered.

Search Strategy

A total of 27 records were initially identified using the Boolean search string in PubMed database:((Rhinology) AND (Artificial Intelligence)) AND (Accuracy). Filters were applied to include only full-text (*n *= 26), English-language (*n* = 26), and human studies (*n *= 17). After applying predefined inclusion criteria (AI application, human study population, diagnostic focus, original research or review format, sample size ≥10, diagnostic application type, full-text availability, and reported diagnostic performance metrics), 12 studies were included in the final synthesis. Five studies were excluded due to insufficient sample size, lack of diagnostic relevance, or absence of performance metrics.

Selection Process

Two reviewers independently screened each paper against inclusion criteria. Studies not meeting methodological or diagnostic performance reporting standards were excluded. Discrepancies were resolved through consensus.

Data Collection

From each included study, the following data were extracted: authors, year, study design, AI technology, clinical application, sample size, comparator, outcomes, and key findings.

Risk of Bias

For primary studies, risk of bias was assessed using a modified version of the Risk of Bias in Non-randomized Studies of Interventions (ROBINS-I) tool. For reviews, the A Measurement Tool to Assess Systematic Reviews 2 (AMSTAR 2) criteria were applied.

Data Synthesis

Given the heterogeneity in design, outcomes, and AI technologies, a narrative synthesis approach was adopted, supported by summary tables of study characteristics and outcomes.

Results

Study Selection and Characteristics

**Figure 1:**
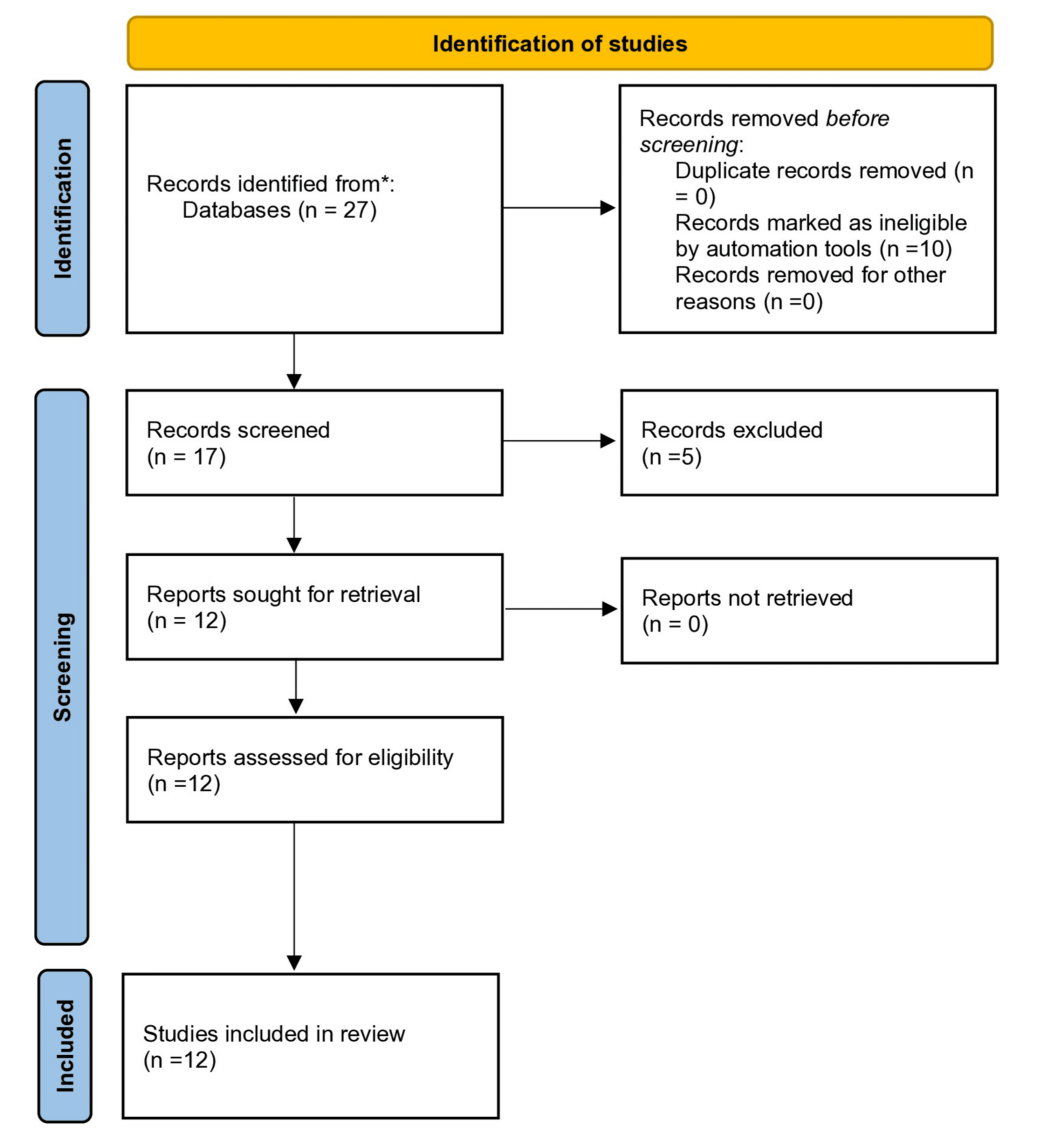
PRISMA flowchart. PRISMA, Preferred Reporting Items for Systematic Reviews and Meta-Analyses

The systematic search identified 27 records, of which 17 underwent screening after initial filtering. Following exclusion of 5 studies due to lack of diagnostic focus or insufficient sample sizes (<10 participants), 12 studies were ultimately included in the final synthesis. The included studies encompassed diverse diagnostic applications of AI in rhinology, comprising six original experimental or observational studies, five reviews including scoping reviews, and one study that evaluated guideline adherence using large language models (Figure 1).

Study Characteristics Summary

The 12 included studies span various diagnostic applications of AI in rhinology. Six were original experimental/observational studies, five were reviews (including scoping reviews), and one study evaluated guideline adherence using large language models. Table 1 presents the data extraction assessment, and Table 2 summarizes the risk-of-bias assessment.

**Table 1:** Data extraction table. CNN, convolutional neural network; KNN, k-nearest neighbors; SVM, support vector machine; RF, random forest; DT, decision tree; LBP, local binary pattern; HOG, histogram of oriented gradients; DNN, deep neural network; AUC, area under the receiver operating characteristic (ROC) curve; LASSO, least absolute shrinkage and selection operator; ML, machine learning; DL, deep learning; ANN, artificial neural network; NLP, natural language processing; FCN, fully convolutional network

S. No.	Author(s) and year	Country	Study design	Population/Sample size	Exposure (AI type)	Comparator/Control	Outcomes (key findings)
1	Ay et al., 2022 [1]	Turkey	Comparative, Experimental	80 subjects; 2,560 polyp images	CNN, KNN, SVM, RF, DT, LBP, HOG	Handcrafted features	CNN: 98.3% accuracy, 99% precision, 98% recall
2	Raghavan et al., 2025 [4]	United States	Observational, Comparative	543 patients	XGBoost, RF, DNN, Logistic Regression	N/A	Accuracy 74.5%-85.5%, low sensitivity (36%-39%), specificity 92%-98%
3	Wong et al., 2020 [2]	Canada	Experimental, Observational	447 CT images	CNN (Inception-V3, transfer learning)	Radiologist	Accuracy 81%, AUC 0.93
4	Dimauro et al., 2019 [3]	Italy	Experimental	12,298 cells	CNN (Python/Keras)	Manual cytology	Accuracy 99% (test), 94% (validation); sensitivity >97%
5	Kaul et al., 2024 [5]	Australia	Review	10 articles	ML (LASSO, ElasticNet, clustering)	Radiologist	Radiomics AUC range: 0.73-0.92
6	Osie et al., 2023 [8]	United States	Scoping Review	79 articles	ML, DL, CNN, ANN, NLP, U-Net, Res-Net	Literature	36.7% rated excellent AUC
7	Wu et al., 2023 [9]	China	Review	78 studies	ML, DL, ResNet50, Xception, InceptionV3, SVM, Hyperspectral Imaging	Literature	AUC 91.9%, mean accuracy 88.4%
8	Ye et al., 2024 [6]	China	Cross-sectional, Comparative	189 AR and 242 CRS questions	ChatGPT (GPT-3.5, GPT-4.0)	Human expert graders	>80% overall accuracy, GPT-4: 74.1% comprehensive
9	Macmath et al., 2023 [10]	United States	Review	Various samples (e.g., asthma, CoFAR2)	ML (SVM, RF, KNN), DL (CNN, RNN), NLP, ANN	Literature	Sensitivity 90.9%, specificity 93.2% (EoE); accuracy up to 97%
10	Tessler et al., 2024 [7]	Israel	Guideline Adherence Assessment	72 ChatGPT responses (24 questions × 3)	ChatGPT 3.5	Expert opinion	59.7% highly accurate, subspecialty accuracy 8%-100%
11	Ramchandani et al., n.d. [11]	United States	Observational, Comparative	350 MCQs (18 image-based)	Gemini, GPT-4, Copilot, Bard	MCQ answer key	Gemini 79.8%, GPT-4 71.1%, Copilot 68.0%, Bard 65.1%
12	Amanian et al., 2024 [12]	United States	Scoping Review	59 articles	ML, DL, FCN, CNN, RF, NLP	Literature	88% (AI vs. junior clinician in AR tool), black-box and data bias limitations noted

**Table 2:** Risk-of-bias assessment. ROBINS-I, Risk of Bias in Non-randomized Studies of Interventions; AMSTAR 2, A Measurement Tool to Assess Systematic Reviews 2

S.No	Author(s)	Risk-of-bias assessment (ROBINS-I /AMSTAR 2)
1	Ay et al., 2022 [1]	Moderate (limited dataset diversity, manual engineering bias)
2	Raghavan et al., 2025 [4]	Serious (low sensitivity, potential overfitting)
3	Wong et al., 2020 [2]	Moderate (small dataset, risk of false negatives)
4	Dimauro et al., 2019 [3]	Low (robust accuracy, but color standardization lacking)
5	Kaul et al., 2024 [5]	Moderate (heterogeneity, Radiomics Quality Score low)
6	Osie et al., 2023 [8]	Low (structured scoping review methodology)
7	Wu et al., 2023 [9]	Low (systematic method, external data needed)
8	Ye et al., 2024 [6]	Moderate (subjective grading, no real-world testing)
9	Macmath et al., 2023 [10]	Moderate (bias and transparency concerns in review)
10	Tessler et al., 2024 [7]	High (small sample, outdated guidelines)
11	Ramchandani et al. [11]	Moderate (non-clinical setting, AI not for standalone use)
12	Amanian et al., 2024 [12]	Low (methodical, but AI black-box and regulation concerns)

Five studies were excluded during the screening stage based on predefined eligibility criteria, which included the following: the study must investigate AI applications specifically in rhinology with a clearly defined methodology; involve human subjects in a clinical setting; evaluate diagnostic accuracy with quantifiable performance metrics such as sensitivity, specificity, accuracy, area under the ROC curve (AUC), or F1 score; and focus primarily on diagnostic applications rather than solely on surgical planning or other non-diagnostic uses. Additionally, the study must be an original research article, systematic review, or meta-analysis with a sample size of at least 10 subjects and be published in the form of a complete research paper rather than an opinion piece, editorial, or conference abstract [13-17].

Diagnostic Performance Across Applications

The included studies demonstrated varying levels of diagnostic accuracy across different rhinological applications. In nasal polyp detection, Ay et al. achieved exceptional performance using convolutional neural networks (CNN), reporting 98.3% accuracy, 99% precision, and 98% recall when analyzing 2,560 polyp images from 80 subjects. Similarly, Dimauro et al. demonstrated outstanding results in cytological analysis, achieving 99% accuracy in testing and 94% in validation phases with sensitivity exceeding 97% across 12,298 cells.

CT analysis for rhinological conditions showed promising but more variable results. Wong et al. reported 81% accuracy with an AUC of 0.93 using CNN-based transfer learning on 447 CT images. In contrast, ML approaches for patient outcome prediction showed more modest performance, with Raghavan et al. reporting accuracy ranging from 74.5% to 85.5% across different algorithms, though these models exhibited concerning low sensitivity (36%-39%) despite high specificity (92%-98%).

Review Study Findings

Multiple systematic and scoping reviews provided broader perspectives on AI applications in rhinology. Wu et al. synthesized 78 studies and reported mean AUC of 91.9% and mean accuracy of 88.4% across various DL and ML approaches. Osie et al. conducted a comprehensive scoping review of 79 articles and found that 36.7% of the studies reported excellent AUC values. Kaul et al. focused specifically on radiomics applications, reporting AUC ranges from 0.73 to 0.92 across 10 articles.

Large Language Model Applications

Emerging applications of large language models in rhinology showed mixed results. Ye et al. evaluated ChatGPT performance on 431 questions related to allergic rhinitis and chronic rhinosinusitis, finding overall accuracy exceeding 80%, with GPT-4 achieving 74.1% comprehensive accuracy. However, Tessler et al. found more concerning results in guideline adherence assessment, with only 59.7% of ChatGPT responses rated as highly accurate, and subspecialty accuracy ranging dramatically from 8% to 100%.

Risk-of-Bias Assessment

Risk of bias varied considerably across studies. Four studies were assessed as having low risk of bias, six demonstrated moderate risk, one had serious risk, and one exhibited high risk. Common concerns included limited dataset diversity, potential overfitting, small sample sizes, and lack of real-world validation. Several studies noted challenges with AI interpretability and regulatory considerations for clinical implementation.

Discussion

This review reveals the diverse and rapidly expanding applications of AI in rhinology, encompassing both traditional diagnostic approaches and emerging technologies. The findings demonstrate significant variation in methodological approaches, diagnostic performance, and clinical readiness across different AI applications in rhinological practice.

Image-Based Diagnostic Applications

The predominance of image-based AI applications reflects the visual nature of rhinological diagnosis and the maturity of computer vision technologies. Convolutional neural networks emerged as the dominant approach for image analysis tasks, consistently demonstrating superior performance compared to traditional ML algorithms. Ay et al. demonstrated the exceptional capability of CNN models in endoscopic nasal polyp detection, achieving 98.3% accuracy, 99% precision, and 98% recall when compared to handcrafted features using traditional algorithms including K-nearest neighbors, support vector machines, and random forest [1]. This performance superiority of DL approaches over conventional ML methods was consistently observed across multiple studies.

Cytological analysis using AI showed particularly promising results, with Dimauro et al. achieving remarkable diagnostic accuracy of 99% in testing and 94% in validation phases using CNN architectures implemented in Python/Keras [3]. The sensitivity exceeding 97% in this application suggests potential for automated screening applications, though the authors noted concerns regarding color standardization that could impact real-world deployment [3]. The robust performance in cytological analysis may be attributed to the standardized nature of cellular imaging compared to more variable endoscopic imaging conditions.

CT analysis presented more heterogeneous results, reflecting the complexity of radiological interpretation. Wong et al. applied transfer learning using Inception-V3 architecture to 447 CT images, achieving 81% accuracy and an AUC of 0.93 when compared to radiologist interpretation [2]. However, the moderate risk-of-bias assessment highlighted concerns regarding small dataset size and potential for false negatives, indicating the need for larger validation cohorts [2]. The radiomics approach reviewed by Kaul et al. demonstrated variable performance with AUC values ranging from 0.73 to 0.92 across 10 articles, emphasizing the heterogeneity in methodological approaches and the impact of feature selection strategies on diagnostic performance [5].

Patient-Reported Outcome Prediction

The application of AI to predict clinical outcomes based on patient-reported measures and clinical variables showed more modest performance compared to image-based applications. Specifically, AI models using patient-reported symptom scores, demographic data, and clinical history were applied to predict chronic rhinosinusitis diagnosis, with accuracies ranging from 74.5% to 85.5%, though sensitivity remained low (36%-39%). Raghavan et al. employed multiple ML algorithms including XGBoost, random forest, deep neural networks, and logistic regression on data from 543 patients, achieving accuracy ranging from 74.5% to 85.5% [4]. However, the concerning finding of low sensitivity (36%-39%) despite high specificity (92%-98%) suggests significant limitations in identifying positive cases, which could have serious clinical implications [4]. The serious risk-of-bias assessment for this study highlighted potential overfitting, a common challenge when applying complex algorithms to relatively small clinical datasets [4].

Large Language Model Applications

The emergence of large language models in rhinology represents a novel frontier with both promising applications and significant limitations. Ye et al. evaluated ChatGPT performance on 431 questions related to allergic rhinitis and chronic rhinosinusitis, demonstrating overall accuracy exceeding 80% with GPT-4 achieving 74.1% comprehensive accuracy when compared to human expert graders [6]. However, the moderate risk-of-bias assessment noted concerns regarding subjective grading criteria and the absence of real-world clinical testing [6].

The comparative evaluation by Ramchandani et al. of multiple large language models, including Gemini, GPT-4, Copilot, and Bard, on 350 multiple-choice questions revealed significant performance differences, with Gemini achieving the highest accuracy at 79.8%, followed by GPT-4 at 71.1%, Copilot at 68.0%, and Bard at 65.1% [11]. Importantly, 18 questions included image-based components, suggesting varying capabilities in multimodal reasoning across different platforms [11].

The guideline adherence assessment by Tessler et al. revealed concerning variability in ChatGPT 3.5 performance, with only 59.7% of responses rated as highly accurate and subspecialty accuracy ranging dramatically from 8% to 100% [7]. The high risk-of-bias assessment for this study noted the small sample size of 72 responses and the use of potentially outdated guidelines, highlighting the challenges in evaluating AI performance against evolving clinical standards [7].

Review Findings

Wu et al. synthesized findings from 78 studies, reporting mean AUC of 91.9% and mean accuracy of 88.4% across various DL and ML approaches including ResNet50, Xception, InceptionV3, support vector machines, and hyperspectral imaging [9]. The low risk-of-bias assessment for this review noted systematic methodology, though external validation data was needed [9].

Osie et al. conducted a comprehensive scoping review of 79 articles examining ML, DL, CNN, artificial neural networks, natural language processing, U-Net, and ResNet applications, finding that 36.7% of studies achieved excellent AUC ratings [8]. The structured scoping review methodology received a low risk-of-bias assessment [8]. Macmath et al. reviewed various samples including asthma and CoFAR2 datasets, reporting sensitivity of 90.9% and specificity of 93.2% for eosinophilic esophagitis detection, with accuracy reaching up to 97% [10]. However, the moderate risk-of-bias assessment highlighted concerns regarding bias and transparency in the review process [10].

Amanian et al. provided insights into comparative performance, noting 88% accuracy when AI was compared to junior clinicians in allergic rhinitis diagnostic tools, while highlighting persistent challenges with black-box AI interpretability and data bias limitations [12]. Despite the methodical approach earning a low risk-of-bias assessment, the authors emphasized ongoing concerns regarding AI explainability and regulatory frameworks [12].

Risk of Bias and Study Quality

The risk-of-bias assessment revealed significant methodological concerns across the included studies. Only four studies (33%) demonstrated low risk of bias, while six studies (50%) showed moderate risk, one study demonstrated serious risk, and one exhibited high risk of bias. Common methodological limitations included small or single-center datasets, lack of external validation, limited dataset diversity, potential overfitting, subjective outcome measures, and absence of prospective validation [1,2,3,4,5,6,7,8,9,10,11,12].

The prevalence of moderate to high risk of bias suggests that while AI applications in rhinology show technical promise, the current evidence base requires strengthening through more rigorous study designs. The concerns regarding dataset diversity and external validation are particularly relevant for clinical translation, as AI models trained on limited or homogeneous datasets may not generalize to broader patient populations or different clinical settings.

Clinical Translation Challenges

Despite the encouraging technical performance metrics reported across studies, a critical finding of this review is the absence of clinically integrated AI tools in routine rhinological practice. This gap between technical achievement and clinical implementation reflects several persistent challenges including regulatory approval processes, integration with existing clinical workflows, cost-effectiveness considerations, and clinician acceptance.

The black-box nature of many DL approaches poses significant challenges for clinical adoption, as healthcare providers require interpretable decision support tools. The concerns raised by multiple authors regarding AI explainability highlight the need for transparent architectures that can provide clinically meaningful insights into diagnostic reasoning [5,6,12].

Future Research Directions

Recent advances in real-time AI applications for rhinology demonstrate the potential for immediate clinical impact through enhanced diagnostic workflows. The development of AI-enabled augmentation systems for nasal endoscopy represents a significant step toward practical clinical integration, with studies showing that edge computing hardware can successfully interface with existing endoscopic equipment while maintaining acceptable performance standards [18]. These real-time applications address one of the key barriers to clinical adoption by seamlessly integrating AI capabilities into established diagnostic procedures without requiring substantial infrastructure modifications. Furthermore, advances in ML approaches for endoscopic image analysis have demonstrated remarkable capabilities in identifying, classifying, and segmenting sinonasal masses, suggesting that AI tools are approaching the sophistication required for complex diagnostic tasks in rhinological practice [19]. The growing emphasis on multimodal AI systems that can process both visual and clinical data simultaneously represents another promising direction, particularly for applications requiring comprehensive diagnostic assessment that incorporates multiple data sources typical of rhinological evaluations [20].

AI applications in rhinology have grown rapidly in recent years, with studies focusing on various aspects such as image processing, diagnostics, and treatment planning [8]. AI has shown promise in improving diagnostic accuracy for sinonasal pathologies, particularly using CT imaging [21]. ML and DL techniques, especially convolutional neural networks, are commonly employed for tasks like classification and segmentation [22,23]. While AI demonstrates potential in enhancing clinical decision-making and personalized medicine in rhinology [24], most current research is retrospective and single-center, limiting generalizability [21]. Future directions include integrating AI with robotics, surgical education, and exploring its role in treatment outcome prediction [25]. Despite promising results, challenges remain in implementing AI in clinical practice, necessitating ongoing validation and multidisciplinary collaboration [26].

The findings of this review underscore several critical priorities for advancing AI applications in rhinology. Multicenter validation studies are essential to address concerns regarding generalizability and external validity. Standardized reporting frameworks specific to AI applications in rhinology would facilitate comparison across studies and improve evidence synthesis. Prospective clinical trials evaluating AI tools in real-world clinical settings are necessary to establish clinical utility beyond technical performance metrics. The integration of AI tools into clinical workflows requires careful consideration of human-AI collaboration models, ensuring that AI enhances rather than replaces clinical expertise. Future research should also address the economic implications of AI implementation, including cost-effectiveness analyses and resource allocation considerations for healthcare systems considering AI adoption in rhinological practice.

## Conclusions

AI holds significant promise for enhancing diagnostic accuracy in rhinology. Applications in image classification, patient-reported outcome prediction, and automated clinical guidance have demonstrated technical feasibility with high accuracy. However, current evidence remains limited to prototype and review stages, with minimal clinical implementation. Rigorous external validation, regulatory alignment, and clinician training are imperative before AI can be safely and effectively integrated into routine ear, nose, and throat (ENT) practice.

## References

[1] Ay B, Turker C, Emre E, Ay K, Aydin G (2022). Automated classification of nasal polyps in endoscopy video-frames using handcrafted and CNN features. Comput Biol Med.

[2] Wong EH, Parmar P, Habib A-R (2020). An artificial intelligence algorithm that identifies middle turbinate pneumatisation (concha bullosa) on sinus computed tomography scans. J Laryngol Otol.

[3] Dimauro G, Ciprandi G, Deperte F, Girardi F, Ladisa E, Latrofa S, Gelardi M (2019). Nasal cytology with deep learning techniques. Int J Med Inform.

[4] Raghavan AM, Aboueisha MA, Prohnitchi I, Cvancara DJ, Humphreys IM, Jafari A, Abuzeid WM (2025). Using machine learning models to diagnose chronic rhinosinusitis: Analysis of pre-treatment patient-generated health data to predict cardinal symptoms and sinonasal inflammation. Am J Rhinol Allergy.

[5] Darbari Kaul R, Sacks PL, Thiel C (2025). Radiomics of the paranasal sinuses: A systematic review of computer-assisted techniques to assess computed tomography radiological data. Am J Rhinol Allergy.

[6] Ye F, Zhang H, Luo X (2024). Evaluating ChatGPT’s performance in answering questions about allergic rhinitis and chronic rhinosinusitis. Otolaryngol Head Neck Surg.

[7] Tessler I, Wolfovitz A, Alon EE (2024). ChatGPT’s adherence to otolaryngology clinical practice guidelines. Eur Arch Otorhinolaryngol.

[8] Osie G, Darbari Kaul R, Alvarado R (2023). A scoping review of artificial intelligence research in rhinology. Am J Rhinol Allergy.

[9] Wu Q, Wang X, Liang G (2023). Advances in image-based artificial intelligence in otorhinolaryngology-head and neck surgery: a review. Otolaryngol Head Neck Surg.

[10] Macmath D, Chen M, Khoury P (2023). Artificial intelligence: exploring the future of innovation in allergy immunology. Curr Allergy Asthma Rep.

[11] Ramchandani R, Guo E, Mostowy M (2025). Comparison of ChatGPT-4, Copilot, Bard and Gemini Ultra on an otolaryngology question bank. Clin Otolaryngol.

[12] Amanian A, Heffernan A, Ishii M, Creighton FX, Thamboo A (2023). The evolution and application of artificial intelligence in rhinology: a state of the art review. Otolaryngol Head Neck Surg.

[13] Grimm DR, Lee YJ, Hu K, Liu L, Garcia O, Balakrishnan K, Ayoub NF (2024). The utility of ChatGPT as a generative medical translator. Eur Arch Otorhinolaryngol.

[14] Fazilat AZ, Brenac C, Kawamoto-Duran D (2025). Evaluating the quality and readability of ChatGPT-generated patient-facing medical information in rhinology. Eur Arch Otorhinolaryngol.

[15] Kim DK, Lim HS, Eun KM (2021). Subepithelial neutrophil infiltration as a predictor of the surgical outcome of chronic rhinosinusitis with nasal polyps. Rhinology.

[16] Lee DJ, Hamghalam M, Wang L (2025). The use of a convolutional neural network to automate radiologic scoring of computed tomography of paranasal sinuses. Biomed Eng Online.

[17] Bellinger JR, Kwak MW, Ramos GA, Mella JS, Mattos JL (2024). Quantitative comparison of chatbots on common rhinology pathologies. Laryngoscope.

[18] Bidwell J, Gyawali D, Morse J, Ganeshan V, Nguyen T, McCoul ED (2025). Real-time augmentation of diagnostic nasal endoscopy video using AI-enabled edge computing. Int Forum Allergy Rhinol.

[19] Levi L, Ye K, Fieux M (2025). Machine learning of endoscopy images to identify, classify, and segment sinonasal masses. Int Forum Allergy Rhinol.

[20] He R, Jie P, Hou W (2025). Real-time artificial intelligence-assisted detection and segmentation of nasopharyngeal carcinoma using multimodal endoscopic data: a multi-center, prospective study. EClinicalMedicine.

[21] Petsiou DP, Spinos D, Martinos A, Muzaffar J, Garas G, Georgalas C (2025). Effectiveness of artificial intelligence in detecting sinonasal pathology using clinical imaging modalities: a systematic review. Rhinology.

[22] Bulfamante AM, Ferella F, Miller AM (2023). Artificial intelligence, machine learning, and deep learning in rhinology: a systematic review. Eur Arch Otorhinolaryngol.

[23] Jun YJ, Jung J, Lee HM (2020). Medical data science in rhinology: background and implications for clinicians. Am J Otolaryngol.

[24] Ayoub NF, Glicksman JT (2024). Artificial intelligence in rhinology. Otolaryngol Clin North Am.

[25] Ghosh Moulic A, Gaurkar SS, Deshmukh PT (2024). Artificial intelligence in otology, rhinology, and laryngology: a narrative review of its current and evolving picture. Cureus.

[26] Tama BA, Kim DH, Kim G, Kim SW, Lee S (2020). Recent advances in the application of artificial intelligence in otorhinolaryngology-head and neck surgery. Clin Exp Otorhinolaryngol.

